# Supplementary material for: TissUUmaps 3: Improvements in interactive visualization, exploration, and quality assessment of large-scale spatial omics data
Source: Heliyon. 2023 Apr 17;9(5):e15306. doi: 10.1016/j.heliyon.2023.e15306 (PMC10149187; doi:10.1016/j.heliyon.2023.e15306)
Supplement: Multimedia component 1 [file mmc1.docx]

**Supplementary Information**

# SI 1 Benchmarking protocols and specifications

## Loading time and memory usage

To measure loading time in the benchmarks in Section 3.2, the following hardware and software specifications were used:

- A simulated, fixed network bandwidth of 100Mbit/s, using a custom Network Throttling Profile of the Chrome developer tools.
- An HTTP server running locally with support for partial requests from the client to remove internet connection variability and allow range access for HDF5 datasets.
- Google Chrome browser v108.0.5359.100 running on Windows 10 64-bit, with an x64-based processor intel Core i7-8650U, 1.90GHz to 2.1GHz, integrated GPU (Intel UHD Graphics 620), and 32 GB of RAM.

Each measure was performed ten times. Time was measured using the JavaScript time function from the start of data downloading until the markers appeared in the viewport. Memory was measured using the JavaScript function performance.memory.usedJSHeapSize in Chrome. The browser tab was closed and reopened between runs to get the most reliable measure of the used heap size. It can be noted that this does not avoid all variations between runs, as the memory usage in JavaScript depends on multiple factors.

The datasets used were sub-sampled randomly from *Vizgen MERFISH FFPE Human Immuno-oncology Data Set, May 2022: Human Ovarian Cancer Patient 2, Slice 1*, with all successive powers of two from 2^6^ to 2^26^.

## Rendering time

To measure rendering time in the benchmarks in Section 3.2, the following hardware and software specifications were used:

- A laptop with an Intel Core i7-6700HQ CPU, integrated GPU (Intel HD Graphics 530), and 32 GB of RAM.
- A desktop with an Intel Core i5-6600K CPU, discrete GPU (NVIDIA RTX 2070 Super, with 8 GB of VRAM), and 32 GB of RAM.
- Google Chrome browser v108.0.5359.98 running on Ubuntu 20.04 64-bit on both computers.

For the WebGL-based marker rendering in TissUUmaps 3, we used timer queries from the API extension EXT disjoint timer query webgl2 to log GPU frame timings for the rasterization of the markers. For the SVG-based marker rendering in TissUUmaps 1, we could not directly measure frame timings in the browser. Instead, frame times were estimated from frame rates captured at different time points via the “Show frames per second (FPS) meter” overlay in the Chrome developer tools.

Average frame time over ten frames was measured for each combination of a dataset (Figure 6) and TissUUmaps version; we also repeated the measurements on both test systems for each rendering method (point sprites or instancing) of TissUUmaps 3. On the test system with the NVIDIA RTX 2070 Super GPU, the performance mode in the NVIDIA X Server Settings control panel was set to “Prefer Maximum Performance” for more consistent frame timings for the smaller datasets. In Figure 6, confidence intervals for the average frame times are also presented.

## Comparison with Vitessce

To measure loading time in the comparison with Vitessce in Section 3.2.1, the following hardware and software specifications were used:

- A laptop with an Intel Core i7-7500U CPU, integrated GPU (Intel HD Graphics 620), and 16 GB of RAM.
- TissUUmaps 3.1.0.
- Vitessce 3.0.3 with the view config schema version set to 1.0.15.

The experiments were conducted using Jupyter Notebook. For TissUUmaps, the timer for the two file formats, CSV and HDF5, was started as soon as the respective functions loaddata() (for CSV) and opentmap() (for H5AD) were called, and manually stopped as soon as markers were displayed in the viewer. For Vitessce, the timer started as soon as the widget() method in the ViewConfig was called, and manually stopped as soon as markers were displayed. The creation of the ViewConfig object was not included in the timing. We used a minimal ViewConfig with only one spatial component, and each measurement was performed three times.

The datasets used were sub-sampled randomly from *Vizgen MERFISH FFPE Human Immuno-oncology Data Set, May 2022: Human Ovarian Cancer Patient 2, Slice 1*, with all successive powers of two from 2^20^ to 2^26^.
